# F81. AGE OF ONSET OF CANNABIS USE AND COGNITIVE FUNCTION IN FIRST EPISODE NON-AFFECTIVE PSYCHOSIS PATIENTS: 3-YEAR FOLLOW-UP OUTCOME

**DOI:** 10.1093/schbul/sby017.612

**Published:** 2018-04-01

**Authors:** Esther Setién-Suero, Diana Tordesillas-Gutierrez, Benedicto Crespo-Facorro, Rosa Ayesa-Arriola

**Affiliations:** 1 Centro de Investigación Biomedica en Red de Salud Mental (CIBERSAM); 2 University Hospital Marques de Valdecilla -IDIVAL, University of Cantabria, CIBERSAM

## Abstract

**Background:**

In recent years, the effect of cannabis use on cognitive functions in patients with psychosis has been widely studied, but results are somewhat contradictory. On the other hand, it has also been studied the relevance of the age of onset of consume, suggesting that the early age of onset of consumption may be related to a greater cognitive impairment.

**Methods:**

349 patients with a first episode of non-affective psychosis were studied. Patients were classified in cannabis users and non-users. Users were divided according to their age at the beginning of use of cannabis in: early-onset (age<16) and late-onset (≥16 years-old). Differences between groups at baseline were studied on sociodemographic, clinical and cognitive variables. The groups were longitudinally (3-year) compared on cognitive variables.

**Results:**

Out of the 349 patients included in this study, 38.7% (N=135) were cannabis users, of them 39.3% (N=53) started consuming before 16 years of age and 60.7% (N=82) did so at age 16 of after. No differences were found between early-onset and late-onset groups on cognitive domains. However, cannabis users (early and late) showed significantly worse performance in processing speed than non-users. Longitudinal analises revealed that the groups of early-onset, late-onset and non-users of cannabis, had different evolution in processing speed domain and in the global cognitive functioning.

**Discussion:**

The main findings of this study were that, although there were differences between patients who used cannabis and those who did not, minimal differences aroused between the early-onset and late-onset cannabis users. With respect to longitudinal analyses, we must be careful with their interpretation, since although a priori we found a significant group by time interaction (early-onset, late-onset, and non-users) in some domain, when the cannabis use at 3-year follow-up was considered, results did not show any significance, this reveals that cannabis users (early-onset and late-onset) and non-cannabis users did not differ in the degree of change in their cognitive functions, regardless of whether or not the patients had maintained consumption during the first 3-year of disease progression.

